# DFT-Net: Deep Feature Transformation Based Network for Object Categorization and Part Segmentation in 3-Dimensional Point Clouds

**DOI:** 10.3390/s22072512

**Published:** 2022-03-25

**Authors:** Mehak Sheikh, Muhammad Adeel Asghar, Ruqia Bibi, Muhammad Noman Malik, Mohammad Shorfuzzaman, Raja Majid Mehmood, Sun-Hee Kim

**Affiliations:** 1 Department of Computer Science, National University of Modern Languages, NUML, Rawalpindi 46000, Pakistan; mehak.sheikh@numl.edu.pk (M.S.); adeel.asghar@numl.edu.pk (M.A.A.); mnauman@numl.edu.pk (M.N.M.); 2 Department of Software Engineering, National University of Modern Languages, NUML, Rawalpindi 46000, Pakistan; ruqiyya.bibi@numl.edu.pk; 3 Department of Computer Science, College of Computers and Information Technology, Taif University, P.O. Box 11099, Taif 21944, Saudi Arabia; m.shorf@tu.edu.sa; 4 Information and Communication Technology Department, School of Electrical and Computer Engineering, Xiamen University Malaysia, Sepang 43900, Malaysia or rajamajid@xmu.edu.my; 5 Department of Brain & Cognitive Engineering, Korea University, Anam-dong, Seongbuk-ku, Seoul 02841, Korea

**Keywords:** point cloud, deep neural network, 3D object categorization, classification, part segmentation

## Abstract

Unlike 2-dimensional (2D) images, direct 3-dimensional (3D) point cloud processing using deep neural network architectures is challenging, mainly due to the lack of explicit neighbor relationships. Many researchers attempt to remedy this by performing an additional voxelization preprocessing step. However, this adds additional computational overhead and introduces quantization error issues, limiting an accurate estimate of the underlying structure of objects that appear in the scene. To this end, in this article, we propose a deep network that can directly consume raw unstructured point clouds to perform object classification and part segmentation. In particular, a Deep Feature Transformation Network (DFT-Net) has been proposed, consisting of a cascading combination of edge convolutions and a feature transformation layer that captures the local geometric features by preserving neighborhood relationships among the points. The proposed network builds a graph in which the edges are dynamically and independently calculated on each layer. To achieve object classification and part segmentation, we ensure point order invariance while conducting network training simultaneously—the evaluation of the proposed network has been carried out on two standard benchmark datasets for object classification and part segmentation. The results were comparable to or better than existing state-of-the-art methodologies. The overall score obtained using the proposed DFT-Net is significantly improved compared to the state-of-the-art methods with the ModelNet40 dataset for object categorization.

## 1. Introduction

Automatic object segmentation and recognition using 3D point clouds is an important and active research area owing to its numerous potentials in a wide range of real-world applications, including 3D reconstruction and modeling, robotics, autonomous navigation, urban planning, disaster management, augmented/virtual reality, surveillance/monitoring, rehabilitation, and many others. These point clouds are produced using a variety of sensors including optical sensors (photogrammetric point clouds) [1,2], time-of-flight sensors, laser scanning or LiDAR (Light Detection Furthermore, Ranging) [3], and more recently using synthetic aperture radars [4,5]. A point cloud essentially comprises of a set of 3D points $\left\{P_{i=1..........n}\right\}$ where every point is characterized by a vector containing cartesian coordinates (*x*, *y*, *z*) of its position in 3D space. In the context of processing 3D point cloud, part segmentation refers to label each point as belonging to a particular class of object, while in recognition/classification, a group of points is assigned a joint label of a certain object category. A recent approach is to divide the point cloud into local neighbors and merge these local features into 1D global descriptors by applying a simple maximum grouping operation where the local features are extracted using MLP and convolutional neural networks. However, the erratic nature of point clouds makes it challenging to find geometry between local adjacent points using fixed-size filters. DFT-Net proposed a new approach to learning more identifiers using a detailed and straightforward coding strategy between adjacent local points to solve this problem. Extends the functional transformation layer with a deep PointNet architecture that directly contains the raw point cloud. This transformation allows us to learn more identifying characteristics by maintaining local neighborhood relationships between each point. In addition, the discriminant function reduces calculation costs and time to improve classification accuracy. Prior to deep learning, a vast body of literature addressed the segmentation/recognition problem in point clouds focused on unsupervised methods (e.g., region-growing [6], clustering [7], edge-based techniques) [8], energy minimization frameworks (e.g., graph cuts) [4], model fitting approaches [9,10] or traditional supervised methods which rely on first extracting 3D handcrafted features (e.g., planar residuals, entropy, eigen-based attributes) within a local spherical neighborhood and later feeding them as input to a supervised classifier (e.g., support vector machine or random forest) for inference. In some approaches (usually graphical models, e.g., random forests [11,12]), the output of the classifier is often coupled (cascaded) with the conditional random field (CRF) to enforce smoothness constraints during inference.

Over the recent years, deep neural networks (DNNs) have outperformed conventional supervised learning methods in solving wide range of computer vision problems. For instance, convolutional neural networks (CNNs) has now become a de facto standard in processing tasks related to scene understanding and image interpretation. Despite of such great successes of DNNs in 2D domain, there has been not much advancement when it comes to 3D point cloud segmentation/recognition. The reason being that point clouds are typically sparse in nature, have varying point density, and are unstructured with no known relationship among neighbors. These factors limit the direct translation of established 2D methods to work over 3D point clouds. To overcome these difficulties, several researchers have proposed to voxelize the 3D point clouds (i.e., make regular/structured 3D voxels or grid cells) to establish a volumetric representation with known neighborhood relationship prior to processing [13,14,15,16,17].

To cope with aforementioned problems, recently researchers have designed DNNs that directly processes the raw unstructured point cloud, i.e., avoid the additional step of voxelization. The pioneering work in this direction is the PointNet, proposed by Qi et al. [18] which proposed a unified architecture for both part segmentation as well as recognition by processing each point independently and subsequently using a simple symmetric function to achieve model invariance with respect to input points permutations. Since it processes each point independently therefore it does not take into consideration the local neighborhood variations. Rather than processing each point independently, few researchers have proposed extensions of PointNet to take into account the fine-grained structural information by applying PointNet over either a nested partitions [19] or the nearest neighbor graph [20] of the input point cloud.

Hua et al. [21] also introduced a point-wise convolution operator that is able to learn point level local features to achieve invariance to point ordering. Similarly, Arshad et al. [22] proposed a cascaded residual network by inducing skip connections in relatively deeper architecture to simultaneous perform semantic point segmentation and object categorization. Although all these networks exploit the local features but does not take into account the geometric relationship/context among the neighboring points and consequently considers points independently while processing at local scale to gain permutation invariance. To capture such a geometric relationship, Wang et al. [23] recently proposed an edge convolution operator that has the ability to learn edge features characterizing the relationship between neighbors and the corresponding point of interest. The integration of basic edge convolution operator with the PointNet architecture shows relatively better results particularly in occluded/cluttered environments. Motivated by this, in this paper we adapt the basic edge convolution operator and augment it with a novel feature transformation layer (FTL) together with a deep PointNet architecture to directly process the raw point clouds. This formation allows to extract more discriminative features by maintaining the local neighborhood relationship among the respective points. The proposed network is robust to outliers and partially handles the gaps in the point cloud data.

The key contributions of the proposed model are as follows:

- The proposed Deep Feature Transformation Network (DFT-Net) consists of a cascading combination of edge convolution and feature transformation layers, capturing local geometric features by preserving adjacent relationships between points.
- DFT-Net guarantees an invariance point order and dynamically calculates the edges in each layer independently.
- DFT-Net can directly process unstructured raw 3D point clouds while achieving part segmentation and object classification simultaneously.
- The DFT-Net evaluation was performed on two standard benchmark datasets: ModelNet40 [24] for object recognition and ShapeNet [24] for part segmentation, and the results are comparable to existing state-of-the-art methods of object recognition.

## 2. Related Work

To directly translate the concepts of 2D DNNs to work with 3D point clouds, several researchers have proposed methods that obtain geometrical neighborhood information by transforming the unstructured point cloud to a structured one by forming a 3D grid or voxels [13,25,26,27,28]. However, such volumetric representation of point cloud data requires more memory as well as additional pre-processing time, and may introduce quantization artifacts limiting the fine-level scene interpretation. To cope with these issues, few researchers have attempted to directly process raw unstructured point clouds. Among them, PointNet [29] has been the pioneering model that directly consumes unstructured input point clouds and classify them into class labels or part labels of that input. An extension to PointNet has been proposed in [19] which hierarchically aggregate local neighborhood points into geometrical features by sampling points and group them into overlapping regions using feature leaning. However, still, it independently treats the points and does not consider any relationship among the point pairs. Li et al. [30], proposed PointCNN architecture that is a generalization of CNN to learn features from point clouds. These point features are extracted from multi-layer perceptrons (MLPs) and are then passed to a hierarchical network where novel *X*-Conv is applied on transformed features. The *X*-Conv is the core of PointCNN that takes neighboring points and features associated as input for convolution [30].

In [31], the authors used a data augmentation framework to apply different augmentation techniques to local neighborhoods. They called this patchAugment. Experimental studies with PointNet++ and DGCNN models demonstrate the effectiveness of PatchAugment for 3D point cloud classification tasks. In the reference, refs. [32,33] the classification of point clouds is carried out with a regularization strategy and a rear projection network, respectively. Both achieved excellent precision to achieve 3D point classification. Hua et al. [21] proposed point-wise CNN architecture where a novel convolutional operator is used to extract point-wise features from each point in the point cloud. The network is practically simple and sorts the input data in a canonical form for recognition task to learn the feature before feeding them into the convolution network [29]. Ref. [22] extended the pointwise CNN by incorporating the residual networks. Yang et al. [34] proposed a novel deep auto encoder called FoldingNet to address processing of point cloud in an unsupervised manner. It has an encoder-decoder formulation where the encoder is essentially a graph based enhancement of pointNet while the decoder does a transformation of a canonical 2D grid onto the 3D surface. Duan et al. [35] proposed a plug-and-play network to explain how the local regions in point clouds are structurally dependent. Unlike PointNet++ which treats the local points individually without considering any interaction among the point pairs. The network concurrently utilize the local information by incorporating their geometrical information with local structures to understand 3D models. Specifically, it captures geometrical and local information for each local region of 3D point cloud. This model is relatively simple and does not require additional computational power.

Yang et al. [36] proposed an end-to-end gumbel subset sampling operation to select the subset of informative input points to learn stronger input representation without having an extra computational cost. Wang et al. [23], also proposed dynamic graph based CNN (DGCNN) model with the use of EdgeConv, which is also an extension of PointNet [29]. They proposed a novel operator to learn geometric features from point cloud that are crucial for the 3D object classification task. A drawback of DGCNN is it only captures the geometrical features among points but they do not maintain local neighborhood relation which consequently results in the loss of semantic context and thus fails to give better output score especially in cluttered scenes. The proposed approach overcomes this limitation by introducing a feature transformation layer (FTL) integrated with an edge convolution layer to independently calculate the edges of each layer to make the points invariant for permutations. The formation of this layer allows to extract more distinctive features by preserving the local neighborhood relationship between each point. The proposed network is robust to outliers and partially addresses gaps in point cloud data. In the next section, we detail the working steps of the proposed network architecture.

## 3. Methodology

### 3.1. Brief Overview

**Figure 1:**
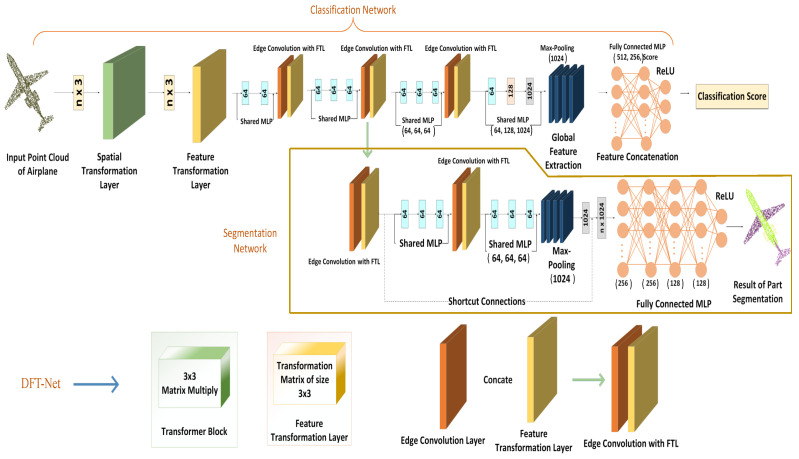
The Proposed Model Architecture.

The proposed network architecture is comprised of classification and segmentation branches and a feature transformation block, as illustrated in Figure 1. The top branch represents the Classification Network and bottom branch represents Segmentation Network. The classification network takes *n* input points, pass to the spatial transformer block to sort the input points then feature transformation layer (FTL) is used to canonicalize the input point clouds. After that edge convolution layer (ECL) uses k-nearest neighbor to calculate the edge features of each layer independently and aggregate these features for corresponding points. The segmentation network extends the classification network. It concatenates global features and the output of all ECLs for every point. Rectified Linear Unit (ReLU) activation function is used with batch normalization function on each layer. The numbers in brackets represent the size of layers. The classification network consumes *n* entry points directly from the point cloud. This entry point *n* is passed to the data-dependent spatial transformer block, which applies the 3×3 matrix to arrange the input in canonical order before departing it to the next function transformer block. The feature transformer block makes points invariant to all types of geometric (such as rotation, translation, scaling, photometric, and affine) transformations which improves the feature learning process and helps the network to perform best in an occluded and noisy environment. On top of it, the proposed model uses local geometrical features by constructing a graph between local neighborhood points and apply edge convolutions with FTL to calculate the edge features on each layer separately. Three-dimensional point clouds are created using directed graphs because they help capture the edges and vertices of the image that are not seen in other graphs. Hence, the graph generated is not fixed and dynamically updated with more discriminative features after every layer. To incorporate the neighborhood context, the *k*-nearest neighbors of a point are utilized, which change from layer to layer to enable the network to learn the features more robustly from a sequence of embeddings [23].

Finally, the final fully connected layer aggregates these optimal features either into a 1D global shape descriptor for the entire image to perform object classification or estimate per point labels for part segmentation. Segmentation networks extend classification networks and merge all ECLs for all points with a one-dimensional global descriptor. The max-pooling layer is used as a symmetric function to add global features. The ECL layer aggregates all the local features together and transform them in to 1D global descriptor. The outcome of the segmentation network is the per-point score for *m* labels.

### 3.2. Edge Convolution with Feature Transformation

Let us assume that the directed graph G∈(V,E) represents the local structure in a point cloud (i.e., points with their *k*-nearest neighbors), where $V=\{V_{1},V_{2},....,V_{n}\}$ represent vertices and $E\subseteq (V_{1}\times V_{n})$ are the edges, respectively. Each of the vertices $\left(V_{1},....,V_{n}\right)$ is the actual 3D point denoted by $\left(x_{1},....,x_{n}\right)$. With this analogy, the graph G is constructed using *k*-nearest neighbors algorithm to find *k* closest neighbors of an individual point $x_{i}$ (i.e., the vertex $V_{i}$). The edge feature is defined by $e_{ij}=h\Theta \left(x_{i},x_{j}\right)$ where Θ represents the set of learnable parameters and $h:R^{F}\times R^{F}\to R^{F^{\prime }}$ is a non-linear function, known as edge function *h*, is parameterized by the set of learnable parameters θ. The edge function *h* and the aggregation operation ∑ has greatly impact on the properties of the resulting Edge Convolution layer (ECL).

**Figure 2:**
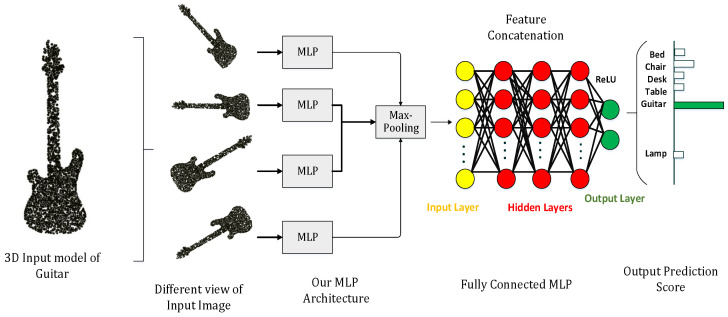
Multi-Layer Perceptron (MLP) with Guitar as an Input.

**Figure 3:**
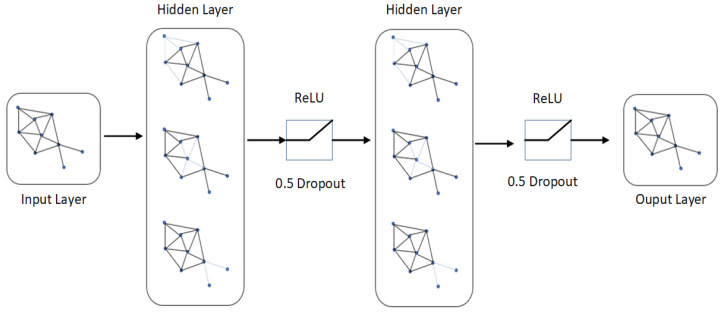
Dynamically update graph on each layer using random dropout which is 0.5 in our case and ReLU as an activation function. The output layer concatenates all the edge features into global shape descriptor.

The FTL is based on multiple multi-layer perceptrons (MLPs), which are used to align the input point clouds in a specific order by maintaining local neighborhood relations among each point. Figure 2 shows how FTL normalizes input functions, redirects input to other MLPs, and groups these input functions through *max-pooling* operations to form more common and recognizable descriptors. The input point cloud of Guitar is passing to the network in multiple angles (different views of same input image), max-pooling is applied to aggregate the features into global descriptors, and these global descriptors are then pass to the final fully connected MLP which will classify it as a guitar by showing maximum output score among all the other classes. The multiple combinations of multilayer perceptrons (MLPs) are used to classify the input point clouds in a specific order while maintaining local neighbor relationships between them. After each perceptron, the maximum grouping is performed to reach the point cloud. A fully connected layer is used to connect the obtained characteristics for all image sets. The input and output layers of the FC layer connect the features in a single row for all images in the dataset. Afterward, The edge convolution with FTL dynamically constructs the graph using *k*-nearest neighbors at each layer of the network to calculate the edge features for the corresponding interest points. The last edge convolution with FTL operation produces output features aggregated into a single 1D global descriptor to generate a classification score of each class. Figure 3 depicts the dynamic graph updation using random dropout and ReLU as an activation function. The edge convolution with FTL is defined by applying symmetric aggregation ∑ operation to concatenate all the edge features from each connected vertex of graph *G*. The output of edge convolution with FTL at *i*-th vertex is

(1) $${x_{i}}^{\prime }=\sum_{j:{(}_{i}{,}_{j})\in \epsilon }h\Theta \left(x_{i},x_{j}\right)$$

In implementation of above, we simply use $h\Theta \left(x_{i},x_{j}\right)=\theta_{j}x_{j}$ with Θ = ($\theta_{i}$....,$\theta_{r}$) representing the weights of the kernel. Since the graph is computed dynamically at each layer, therefore the output of the *m*-th layer is computed as

(2) $$x_{i}^{\left(m+1\right)}=\sum_{j:{(}_{i}{,}_{j})\in \epsilon^{\left(m\right)}}h\Theta^{\left(m\right)}\left(x_{i}^{\left(m\right)},x_{j}^{\left(m\right)}\right)$$

where $x_{j_{i_{1}}}^{\left(m\right)},.......,x_{j_{i_{rm}}}^{\left(m\right)}$ are the nearest points to $x_{i}^{\left(m\right)}$.

### 3.3. Proposed Network Architecture

#### 3.3.1. Object Categorization

As depicted in Figure 1 (Top branch), the classification network takes *n* input points from the point cloud that is passed to a spatial transformer block to sort the input. FTL then normalizes the input point cloud and makes the points for various geometric and photometric transformations to extract more distinctive features. The FTL has two shared fully connected (64,64) layers followed by three edge convolutions coupled with FTLs. The first two edge convolution layers with FTL have three shared fully connected (64,64,64) layers that help extract geometrical features. The third edge convolution with FTL shares a fully connected layer (128). We also add shortcut connections to remove multi-scale features, and one shared fully connected layer (1024) aggregates these scale point-wise features into higher dimensionality. We use a max-pooling function to get global features. Two multi-layer perceptrons (512,256) are used to aggregate all local and international point information and align input points, and point features together.

#### 3.3.2. Part Segmentation

The bottom branch of Figure 1 represents the part segmentation network which extends the classification network by concatenating 1-D global descriptor and output of all the edge convolution layers for every point. The outcome of the segmentation network is the per-point score for *m* labels. The proposed network uses MLPs to perform part segmentation. It uses a spatial transformer block with one feature transformation layer having two shared fully connected layers (64,64), three edge convolution layers with FTLs having nine shared fully connected layers (64,64,64,64,64,64,64,64,64). Each edge convolution with FTL has three shared fully connected layers (64,64,64) and one shared fully connected layer (1024), which aggregate all the information from previous layers and transform pointwise features into higher dimensionality features. Furthermore, We also included shortcut connections to all edge convolutions with FTLs to extract multi-scale features that output as local feature descriptors. We used a max-pooling function with four multi-layer perceptrons (256, 256, 128, 128) to accumulate and transform local pointwise features into the global descriptor.

## 4. Implementation Details

### 4.1. Network Training

For both the object classification and part segmentation, we follow the same strategy that is used in [23]. i.e., we used the learning rate of 0.001, which is divided by 2 for every 20 epoch. The initial decay rate for batch normalization is set to 0.5, which goes to the final value of 0.99. The whole architecture has been trained using stochastic gradient descent with momentum. The used batch size is set to 32, while the used value for momentum is 0.9. ReLU activation function has been used, and batch normalization is used in all the layers. For classification/categorization purposes, the value of *k* in *k*-nearest neighbor is set to 20. The dropout of 0.5 is used in the last two layers, while for part segmentation, the used value of *k* is 25 due to the increase in the point density.

### 4.2. Training Time and Hardware

The proposed DFT-Net model takes almost 20–24 h to train 100 iterations of the base network until it converges (usually takes 20 epochs) when trained from scratch. These timings are estimated using the aforementioned parameters using a batch size of 32 while carrying out all the training on a single Tesla K80 GPU equipped desktop computer with the following details: Intel(R) Xeon(R) CPU E5-2620 v4 2.10 GHz and 16 GB RAM. For both the object categorization and part segmentation, the total GPU memory consumed is around 12 GB. These estimates can easily be improved using a multi GPU configuration.

The computational cost or loss of depth information is calculated separately for each set of images. 3D Point cloud segmentation is the process of classifying point clouds into different homogeneous regions so that points in the same isolated and significant area have similar properties.

(Repository Detail) Data folder: Contains the dataset ModelNet40 that can be downloaded (//Download HDF5 of ModelNet40 for Object recognition dataset (around 450 MB)).

*Model folder:* In model folder we have two separate files. DFT-Net contains all implementation details of our object categorization model. It defines what features are we extracted and how many neurons are being used in each layer. How Max-pooling applied and how to get the categorization score.

The transform1.net file gives the implementation details about how ECL combines with feature transformation layer and update graph dynamically on each layer. The Part Segmentation folder contains details regarding to part segmentation. The dataset we used, training and testing of data and GPU’s regarding details.

## 5. Experimental Evaluation

This section analyzes the constructed model using edge convolutions with FTL for specific tasks, including object recognition and part segmentation. We also evaluate the experimental results by illustrating the key differences from previous models.

### 5.1. Materials

For classification, the ModelNet40 [24] dataset has been used for evaluation. The dataset has 13,311 CAD models from 40 object categories. This dataset is split into 9843 models for training and 2468 models for testing. We directly work on raw point clouds, whereas previously, all methods focused on volumetric grids and multi-view image processing. We sampled 1024 points uniformly from meshes and normalized these points into a unit sphere. The (x,y,z) coordinates from the sampled point clouds are used while original meshes are discarded. During the training, the processed data is augmented by randomly rotating and scaling the object position of every point using Gaussian noise having zero mean and 0.02 standard deviation.

**Figure 4:**
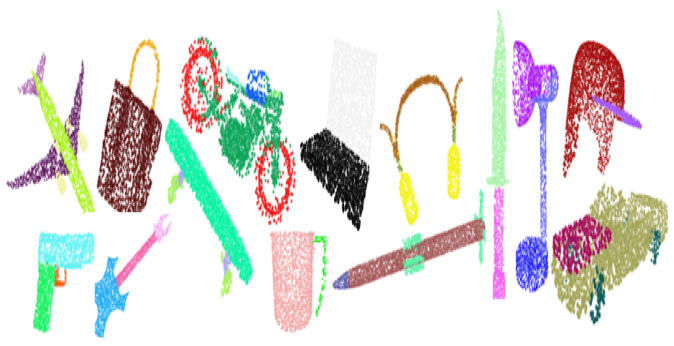
Qualitative analysis of part segmentation result on complete input dataset. We visualize the part segmentation result on ShapeNet part (CAD Models), having 16 different object categories. The visualization result shows that our network performs better on a benchmark dataset.

Part segmentation is one of the most challenging 3D recognition tasks. We extended the proposed classification model to part segmentation on ShapeNet part dataset [24]. The main task of part segmentation is to assign a new predefined part label (e.g., laptop lid, car tire, etc.) to every point of a point cloud. Figure 4 quantitatively analyze the result of the part segmentation model on the ShapeNet part dataset, which consists of 16,881 3D CAD models from 16 categories, annotated with a total of 50 parts. For every training example, 2048 points are sampled, and the most sampled objects are labeled between two to five elements. We used the public train/test split in our experiments.

### 5.2. Object Categorization Results

Table 1 shows the average-class as well as the overall recognition/categorization results achieved on ModelNet40. The data is divided into a training set and a test set. Average class accuracy is the average of each accuracy per class (sum of the predicted accuracies for each class/number of classes), overall accuracy, number of correctly predicted items/sum of predictable items. In other words, the average class accuracy is the accuracy of a specific image of a set of images in the dataset, and the overall classification accuracy is the accuracy of all images in the dataset, as shown in Figure 4.

**Table 1:** Overall and average-class accuracies achieved on ModelNet40 dataset.

Methods	Avg. Class Accuracy	Overall Accuracy
3D ShapeNets [24]	77.3	84.7
VoxNet [13]	83.0	85.9
Subvolumes [37]	86.0	89.2
Pointwise Convolution [21]	81.4	86.1
ECC [38]	83.2	87.4
Learning SO(3) [39]	86.9	88.9
DPRNet 8-Layers [22]	81.9	86.1
DPRNet 16-Layers [22]	82.1	85.4
Spherical CNN [40]	85.2	89.7
PointNet [29]	86.0	89.2
DGCNN [23]	88.8	91.2
kD-Net [41]	-	90.6
MRTNet-VAE [42]	-	86.4
3DContextNet [43]	-	91.1
FoldingNet [34]	-	88.4
LearningRepresentations [44]	-	84.5
SRN-PointNet++ [35]	-	91.5
PAT (GSA only) [36]	-	91.3
PAT (FPS) [36]	-	91.4
PAT (FPS + GSS) [36]	-	91.7
LightNet [45]	-	86.9
PointNet++ [19]	-	90.7
FusionNet [27]	-	90.8
**DFT-Net**	**90.1**	**92.9**

Similarly, Table 2 shows the effect of varying *k* on the overall model accuracy. As can be seen that the proposed DFT-Net has better accuracies as compared to current state-of-the-art models primarily due to the fact that the edge convolution with FTL dynamically updates the graph using *k* nearest neighbor at every layer. In contrast, the PointNet [29], PointNet++ [19], MoNet [46] and other graph-based CNN architectures work with a fixed graph on which convolution operations are applied.

For instance, in PointNet *k* = 1, which shows that the graph is not dynamically updated on each layer, resulting in an empty edge graph formation at each layer. This indicates that the edge function used in PointNet only considers the global geometry of points and ignores the local geometry. The aggregation operation ∑ is used, which is a single node operation in PointNet. PointNet++ overcome the drawback of PointNet by constructing a graph using Euclidean distances between each layer of points through coarsening operation on graph edges. At each layer of PointNet++, some points are selected based on the farthest point sampling algorithm. These selected points get retained while others are discarded at each layer; hence the graph becomes smaller after applying operation on each layer separately. PointNet++ also uses the ∑ as aggregation operation. The main difference between the DFT-Net model approach with others is that we use the feature transformation layer with the edge convolution block. This feature transformation layer aligns the input points in sequential order and is invariant to different transformations. The edge convolutions with FTL dynamically update the graph on each layer, enabling learning the local features and maintaining the geometric relationship among the points of the point cloud, consequently resulting in better overall accuracy.

### 5.3. Part Segmentation Results

We evaluate our model using the standard evaluation strategy, i.e., we used intersection over union (IoU) to compare our result with other benchmarks. The IoU of a shape is computed by taking an average of different parts of IoU that occur in shape. The mean IoU (mIoU) is obtained by taking an average of IoUs of all testing shapes. Table 3 and Table 4 provides the overall and class-wise accuracies, respectively, which are also competitive (slightly better) in comparison to existing state-of-the-art models.

**Table 3:** Overall Accuracy of Part Segmentation Results on ShapNet Part dataset.

Methods	Overall Accuracy
PointNet++ [19]	85.1
KD-Tree [41]	82.3
FPNN [47]	81.4
SSCNN [48]	84.7
PointNet [29]	83.7
LocalFeature [49]	84.3
DGCNN [23]	85.1
FCPN [50]	84.0
RSNet [51]	84.9
ASIS (PN) [52]	84.0
ASIS (PN++) [52]	85.0
**DFT-Net**	**85.2**

Figure 4 shows the qualitative results of part segmentation achieved on the ShapeNet dataset on different object categories.

### 5.4. Model Robustness

**Figure 5:**
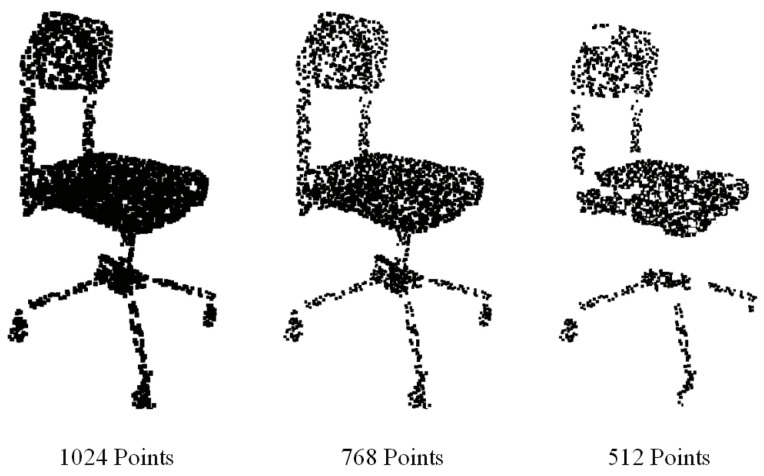
Effect on accuracy with reduced point clouds.

**Figure 6:**
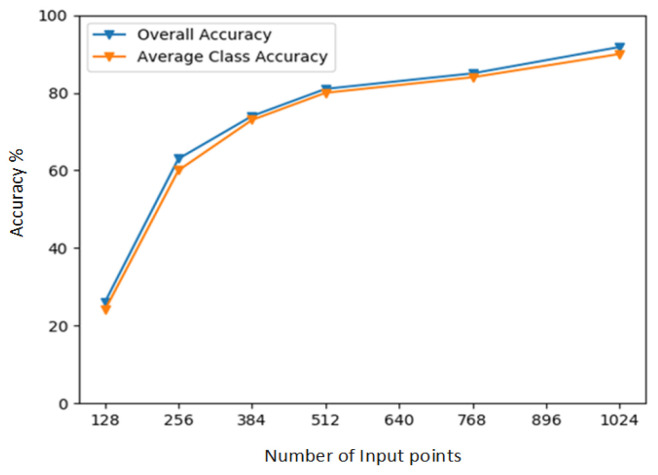
The graph of Figure 5 is depicted in Figure 6. The Curve in a graph represents the testing result of DFT-Net. The final model is trained with 1024 number of points having *k* = 20.

Figure 5 shows an example of DFT-Net performance on partial point cloud on one object category. As evident, the DFT-Net performs reasonably well by dropping half of the points in the point cloud. *Left:* To verify the robustness of the proposed algorithm, we have randomly reduced the number of points and fed the obtained partial point cloud to the object categorization module. Initially in ModelNet40, every object contains 1024 points (*the left point cloud*). We reduced them to 768 (*middle*) and 512 (*right*) partial point clouds and obtained the correct inference using them. In Figure 6, the curves in a graph depict the testing accuracy of the classification/categorization model of DFT-Net. The model is trained with 1024 points. However, the performance degenerates with lesser than 384 points. Figure 6 contains all sample images with different input point clouds, overall classification accuracy compared to class average performance. The curves on the graph represent the accuracy of the DFT-Net classification model test. All images are used to calculate the point cloud with the same characteristics. The DFT-Net model was also trained by adding Gaussian noise and salt and pepper noise, but the model did not perform well. However, the DFT-Net model offers better results than many modern models while adding noise.

**Figure 7:**
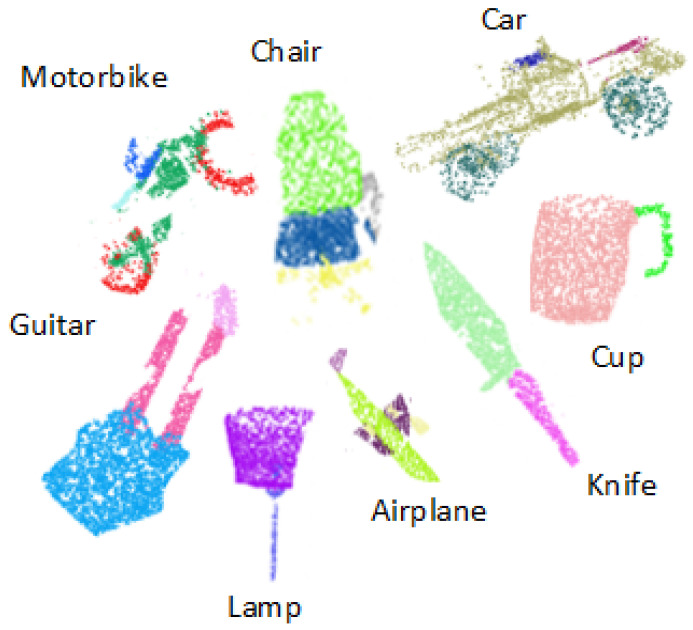
Qualitatively the result is visualized with 16 different object categories, shows that our network performs state-of-the-art in the partial input dataset.

**Figure 8:**
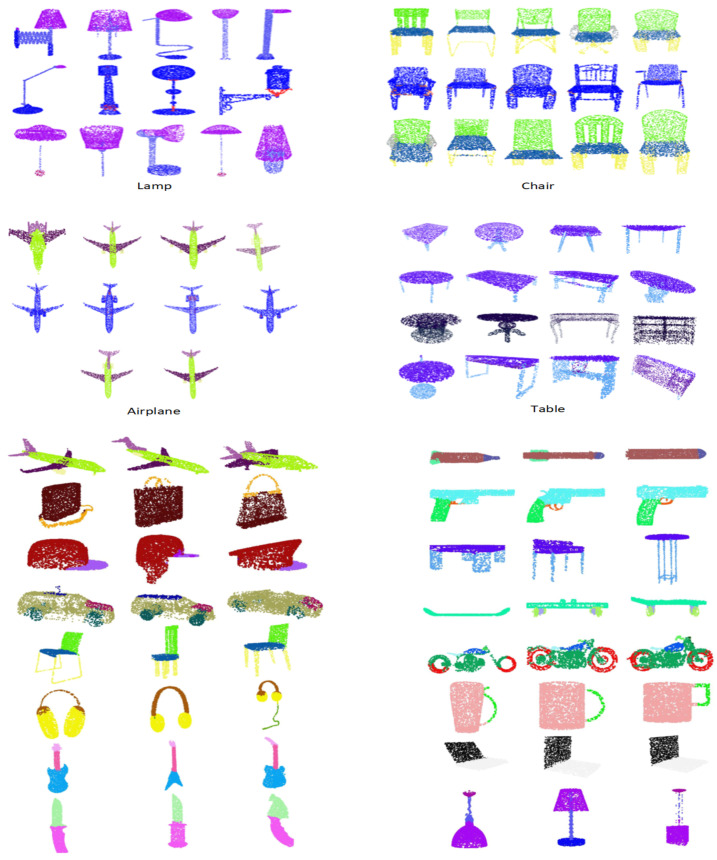
Results of part segmentation on different object categories.

The authors of [53] have also proposed calculating 3D points using partial image sets. The proposed DFT-Net outperforms the previous models based on multi-view and volumetric grid architectures on the complete dataset. To further validate the robustness of the model, we also evaluated the performance of the DFT-Net on the partial dataset. For this purpose, we reduced the number of points in the input point cloud during the testing phase. The DFT-Net model is invariant for all types of photometric and geometric transformations. Therefore, the model offers the best results, even for partial images. The shapes are removed in a specific proportion, and then the shapes are recreated. Figure 7 shows a visualization of DFT-Net results obtained from a partial subset of ShapeNet data for some categories of objects, demonstrating the robustness of DFT-Net even at low point densities. The partial data set is considered the worst case with an overall classification accuracy of 76.4%, as shown in Figure 7. When training DFT-Net, the data si also trained by adding Gaussian noise and salt-n-pepper noise, but the performance of the model was not good. However, our DFT-Net model gives better results than many modern models available, while adding noise. Figure 8 depicts the part segmentation results on different instances of the same individual categories.

We also performed a similar experiment for part segmentation, where we regenerated the shapes by dropping every shape with specific ratios. Similarly, the visualization of DFT-Net results achieved on partial ShapeNet Part dataset over some object categories is depicted in Figure 7 which shows the robustness of DFT-Net even with low point density.

## 6. Conclusions and Future Work

This paper proposed a deep learning architecture to perform 3D object recognition and part segmentation simultaneously. The proposed network directly consumes the raw input points from the point cloud without any conversion into an intermediate voxel space. DFT-Net helps create 3D point clouds by maintaining the local neighbor relationships of the point clouds. It is also very difficult to learn discriminant functions directly from point clouds. The proposed DFT-Net model is very useful to learn more discriminant features using a simple and detailed encoding strategy between local adjacent points. In addition, the discriminant features reduce calculation costs and time to improve classification accuracy. The proposed model achieves an overall classification performance of 85.2% with the ShapeNet Dataset and 92.9% with the ModelNet40 dataset, much better than other modern models at present. The proposed model’s better performance compared to other state-of-the-art methods is essentially based on adding a feature transformation layer that makes points invariant to all transformations and helps extract more discriminative features from the feature space. With this regard, our model also suggests the equal distribution of intrinsic and extrinsic features to create a balancing pipeline to process geometrical features. DFT-Net model is invariant for all kinds of photometric and geometric transformations. Therefore, the proposed model gives the best results, even for partial images.

Although the proposed network performs well, few points are worth mentioning and may be considered in further extension of this work:

- *Fusion of Non-Spatial Attributes*: In the proposed model, we only considered 3D coordinates $x=(x_{i},y_{j},z_{k})$ of points to perform object categorization and part segmentation. Another exciting work might be to fuse traditional hand-crafted point cloud features (e.g., color, point density, surface normals, texture, curvature, etc.) together with extracted deep features or spatial coordinates for enhanced feature representation, consequently improve model performance.
- *Distance-Based Neighborhood*: Instead of capturing local relationships to build a dynamic graph, the distance-based neighbors may be used instead of *k*-nearest neighbors. This may allow for incorporating the semantics of being physically close in selecting the neighbors of the point of interest;
- *Non-Shared Feature Transformer*: Another extension would be to design a non-shared feature transformer network that may work on the local patch level, consequently adding more flexibility to the proposed model.

## Figures and Tables

**Table 2:** Classification accuracy of our model using different *k*-nearest neighbors.

Different Values of *k*-Nearest Neighbors	Average Class Accuracy	Overall Accuracy
10	87.5%	89.2%
15	89.2%	91.5%
20	90.1%	92.9%
25	88.2%	91.2%
30	88.6%	91.2%
35	80.3%	89.2%
40	80.0%	85.2%

**Table 4:** Results of Part Segmentation on ShapeNet part Dataset.

Methods	Aero	Bag	Cap	Car	Chair	Guitar	Knife
No. of Shapes	2690	76	55	898	3758	787	392
PointNet++ [19]	82.4	79.0	87.7	77.3	90.8	91.0	85.9
KD-Tree [41]	80.1	74.6	74.3	70.3	88.6	90.2	87.2
FPNN [47]	81.0	78.4	77.7	75.7	87.6	92.0	85.4
SSCNN [48]	81.6	81.7	81.9	75.2	90.2	93.0	86.1
PointNet [29]	83.4	78.7	82.5	74.9	89.6	91.5	85.9
LocalFeature [49]	86.1	73.0	54.9	77.4	88.8	90.6	86.5
DGCNN [23]	84.2	83.7	84.4	77.1	90.9	91.5	87.3
FCPN [50]	84.0	82.8	86.4	88.3	83.3	93.4	87.4
RSNet [51]	82.7	86.4	84.1	78.2	90.4	91.4	87.0
**DFT-Net**	**97.0**	**99.2**	**98.4**	**97.7**	**99.1**	**96.0**	**99.7**
**Methods**	Lamp	Laptop	Bike	Mug	Pistol	Table	Skate Board
No. of Shapes	1547	451	202	184	283	5271	152
PointNet++ [19]	83.7	95.3	71.6	94.1	81.3	82.6	76.4
KD-Tree [41]	81.0	94.9	57.4	86.7	78.1	80.3	69.9
FPNN [47]	82.5	95.7	70.6	91.9	85.9	75.3	69.8
SSCNN [48]	84.7	95.6	66.7	92.7	81.6	82.1	82.9
PointNet [29]	80.8	95.3	65.2	93.0	81.2	80.6	72.8
LocalFeature [49]	75.2	96.1	57.3	91.7	83.1	83.8	72.5
DGCNN [23]	82.9	96.0	67.8	93.3	82.6	82.0	75.5
FCPN [50]	77.4	97.7	81.4	95.8	87.7	73.4	83.6
RSNet [51]	83.5	95.4	66.0	92.6	81.8	82.2	75.8
**DFT-Net**	**99.7**	**99.7**	**89.5**	**99.6**	**97.9**	**99.8**	**100**

## Data Availability

Not Applicable.

## Link to the Code

The code and the achieved results have been made public at the following link: https://github.com/Mehakxheikh/DFT-Net.

## Abbreviations

The following abbreviations are used in this manuscript:

2D	2-Dimensional
3D	3-Dimensional
CNN	Convolutional Neural Networks
CRF	Conditional Random Field
DFT-Net	Deep Feature Transformation Network
DGCNN	Dynamic Grapgh Convolutional Neural Networks
DNN	Deep neural Networks
ECL	Edge Convolutional Layer
FTL	Feature Transformation Layer
IoU	Intersection over Union
LiDAR	Light Detection Furthermore, Ranging
mIoU	mean Intersection over Union
MLP	Multi-Layer Perceptrons
ReLU	Rectified Linear Unit
